# Diagnostik und Therapie der Membranösen Nephropathie – 2023

**DOI:** 10.1007/s00508-023-02261-w

**Published:** 2023-09-20

**Authors:** Marcus D. Säemann, Balazs Odler, Martin Windpessl, Heinz Regele, Kathrin Eller, Irmgard Neumann, Michael Rudnicki, Philipp Gauckler, Andreas Kronbichler, Maarten Knechtelsdorfer

**Affiliations:** 1 6. Medizinische Abteilung mit Nephrologie & Dialyse, Klinik Ottakring, Wien, Österreich; 2 Medizinische Fakultät, SFU, Wien, Österreich; 3 Klinische Abteilung für Nephrologie, Abteilung für Innere Medizin III (Nephrologie, Dialyse und Hypertensiologie), Medizinische Universität Graz, Graz, Österreich; 4 Abteilung für Innere Medizin IV, Klinikum Wels-Grieskirchen, Wels, Österreich; 5 Medizinische Fakultät, JKU, Linz, Österreich; 6 Klinisches Institut für Pathologie, Medizinische Universität Wien, Wien, Österreich; 7 Vasculitis.at, Wien, Österreich; 8 Immunologiezentrum Zürich (IZZ), Zürich, Schweiz; 9 Department Innere Medizin IV (Nephrologie und Hypertensiologie), Medizinische Universität Innsbruck, Innsbruck, Österreich

**Keywords:** Nephrotisches Syndrom, Primäre membranöse Nephropathie, PLA2-Rezeptor-Autoantikörper, IgG-Subtyp 4, SGLT-2-Inhibitor, Nephrotic syndrome, Primary membranous Nephropathy, PLA2-receptor autoantibodies, IgG-subtype 4, SGLT-2-inhibitor

## Abstract

Die membranöse Nephropathie (MN) ist eine Immunkomplex-Glomerulonephritis und zählt zu den häufigsten Ursachen für ein nephrotisches Syndrom beim Erwachsenen und zählt zu den autoimmunen Nierenerkrankungen mit der höchsten Rate an Spontanremissionen. Das häufigste Autoantigen (> 70 % der Fälle) ist gegen den Phospholipase-A2-Rezeptor (PLA2-R) gerichtet und erlaubt mit seinem Nachweis und Verlauf eine hervorragende Diagnostik sowie auch ein optimales Therapiemonitoring. Andere Autoantigene werden laufend veröffentlicht und werden künftig einen autoantigen-basierten Diagnose- und Therapiealgorithmus der MN ermöglichen. Bei fehlender Spontanremission stellt eine spezifische B‑Zell-gerichtete Therapie, insbesondere mit Rituximab die initiale Therapie der Wahl dar. Kalzineurin-Inhibitoren oder Cyclophosphamid sollen erst bei sorgsamer Indikation im jeweiligen klinischen Kontext wie bei ernsthaften klinischen Konsequenzen sowohl durch das nephrotische Syndrom als auch bei Nierenfunktionsverlust erwogen werden. Da renale Immunkomplexe oft lange Zeit benötigen, um abgebaut zu werden, kann eine große Proteinurie der immunologischen Remission durchaus über viele Monate hinterherlaufen, bis es schließlich zu einer Abnahme oder Resolution der Proteinurie kommt. Die Therapie der MN stellt den günstigen Fall einer präzisionsmedizinisch-basierten Therapie in der Nephrologie dar, wobei neue therapeutische B‑Zellantikörper für die seltenen, aber schwierigen Verlaufsformen der MN in naher Zukunft Eingang in die klinische Routine finden werden.

## Abstract (en)

Membranous nephropathy (MN) is an immune-complex glomerulonephritis and is one of the most common causes of nephrotic syndrome in adults and is also one of the autoimmune kidney diseases with the highest rate of spontaneous remission. The most common autoantigen (> 70% of cases) is directed against the phospholipase A2 receptor (PLA2-R) and, with its detection and clinical course, allows for excellent diagnostics as well as optimal therapy monitoring. Other autoantigens are constantly being published and will enable an autoantigen-based diagnostic and therapeutic algorithm for MN in the future. In the absence of spontaneous remission, a specific B‑cell-directed therapy, especially with rituximab, is the initial therapy of choice. Calcineurininhibitors or cyclophosphamide should only be used if they are carefully indicated in the respective clinical context and if there are serious clinical consequences both from the nephrotic syndrome and from loss of kidney function. Since immune complexes within the kidney often require a long time to be degraded, proteinuria response can follow the immunological remission after many months. The therapy of MN represents the favorable case of a precision medicine-based therapy in nephrology, whereby new therapeutic B‑cell antibodies for the rare but difficult forms of MN will find their way into clinical routine in the not-too-distant future.

## Hintergrund

Die membranöse Nephropathie (MN) ist mit einem Anteil von 30 % eine der häufigsten Ursachen für ein nephrotisches Syndrom beim Erwachsenen. Die Inzidenz beträgt 1:100.000, der Erkrankungsgipfel liegt in der 4. bis 5. Lebensdekade, Männer sind häufiger betroffen als Frauen [2:1].

Die MN gehört zu den autoimmunen Nierenerkrankungen mit der höchsten Rate an Spontanremissionen, welche in ungefähr einem Drittel der Fälle auftritt. Bei den zwei Dritteln der Patienten ohne Spontanremission besteht bei der Hälfte eine persistierende Proteinurie bei über langer Zeit erhaltender Nierenfunktion, die andere Hälfte schreitet rascher zum terminalen Nierenversagen fort.

Sollte es nicht zu einer Spontanremission kommen, steht aus therapeutischer Sicht bei den primären Formen eine immunsuppressive Therapie, bei den sekundären Formen, z. B. im Rahmen von Infektionen oder Malignomen, die Behandlung der Grunderkrankung im Vordergrund. Nach Nierentransplantation kommt es in etwa 40 % der Fälle zu einer Rekurrenz der Grunderkrankung.

## Ätiologie und Einteilung

**Figure 1:**
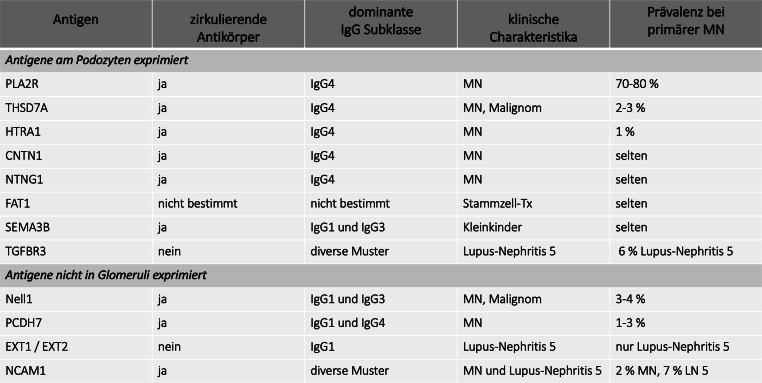

Die MN ist eine Immunkomplex-Glomerulonephritis. Die häufigsten Autoantigene, richten sich gegen den Phospholipase-A2-Rezeptor (PLA2R) und gegen Thrombospondin type‑1 domain-containing protein 7A (THSD7A), welche physiologisch in den Podozyten exprimiert werden [1]. In den letzten Jahren sind mehrere neue Autoantigene entdeckt worden, manche davon werden allerdings nicht in den Glomeruli exprimiert, und deren routinemäßige Bestimmung ist derzeit nicht möglich (siehe Abb. 1). Weitere Antigene werden fortlaufend identifiziert, deren pathophysiologische Bedeutung in der Genese der MN derzeit aber z. T. noch unklar ist.

Histopathologisch ist die MN durch subepitheliale (epimembranöse) Immunkomplex-Ablagerungen charakterisiert, welche in der Immunhistochemie (IHC)/-fluoreszenz (IF) ein typisches diffuses granuläres Muster entlang der Basalmembranen zeigt. Es handelt sich um Komplexe mit Immunglobulin G, welche bei den häufigsten Autoantigenen PLA2R und THSD7A v. a. IgG-Subtyp 4 dominant sind. Je nach Autoantigen können auch andere IgG Subtypen (IgG 1 und 3) dominieren (siehe Abb. 1). Immunkomplexe aktivieren lokal den klassischen und alternativen Komplementweg und führen damit zu nachweisbaren C3/C4-Ablagerungen (IHC/IF), welche morphologische sowie funktionelle Veränderungen der Podozyten bedingt.

Derzeit wird bei der MN noch zwischen einer primären und einer sekundären Form unterschieden. Vermutlich wird aber in Zukunft die MN durch serologische Autoantikörperbestimmungen bzw. Nachweis des Zielantigens in den abgelagerten Immunkomplexen in der Nierenbiopsie besser charakterisiert und auch eingeteilt werden [2].

Wir empfehlen aber bereits jetzt bei PLA2R-Ak-Nachweis im Serum und/oder immunhistochemisch/IF positives PLA2R-Antigen (PLA2R-Ag) in den abgelagerten Immunkomplexen in der Nierenbiopsie, von einer *PLA2R-Ak-assoziierten MN* und entsprechend bei z. B. THSD7A-Nachweis von einer *THSD7A-Ak-assoziierten MN* zu sprechen. Bestimmungen der PLA2R-Ak im Serum sowie Nachweis des PLA2R-Antigens in der Nierenbiopsie gehören zu Standard-Untersuchungen, wobei die Bestimmung der anderen Antigene bis dato nur in spezialisierten Laboren stattfindet. Bei primärer MN können in ca. 75 % der Fälle PLA2R-Ak und in 5–10 % THSD7A-AK (bei PLA2R-Ak-negativen Patienten) nachgewiesen werden.

Eine „primäre“ MN liegt in 70–80 % der Fälle vor, die 20–30 % der „sekundären“ MN-Fälle werden durch Infektionen (z. B. Hepatitis B und selten Hepatitis C, Syphilis), Malignome (meist solide Tumoren aus Prostata, Lunge, Gastrointestinaltrakt, seltener hämatologische Neoplasien wie chronisch-lymphatische Leukämie), Medikamente (z. B. NSAR, Gold, Penicillamin), systemische Autoimmunerkrankungen (z. B. systemischer Lupus erythematodes, rheumatoide Arthritis) und verschiedene Erkrankungen wie z. B. Sarkoidose und Graft-vs.-Host-Disease verursacht [2].

Die MN folgt nicht einer Mendelschen Vererbung, es gibt jedoch Assoziationen zu Mutationen in HLA-DQA1- und PLA2R-kodierenden Genen [1].

## Klinik

Bei 60–80 % aller MN Patienten besteht bei Diagnosestellung ein nephrotisches Syndrom, bei den übrigen Patienten eine nicht-nephrotische Proteinurie, welche bei Nicht-Therapie in ca. 60 % der Fälle im Verlauf zu einem nephrotischen Syndrom fortschreitet. Eine Mikrohämaturie kommt in etwa 30–40 % der Fälle vor, Erythrozytenzylinder sind jedoch sehr selten. Die Nierenfunktion ist bei 80 % normal oder nur geringgradig eingeschränkt.

Insgesamt stellt die MN trotz oft eindrucksvoller Klinik des nephrotischen Syndroms meist keine akut lebensbedrohliche oder die Nierenfunktion akut gefährdende Erkrankung dar. Die möglichen Komplikationen des nephrotischen Syndroms wie z. B. Thromboembolien, die Lebensqualität-Einschränkung durch die nicht selten ausgeprägten Ödeme sowie die Gefahr eines akuten Nierenversagens im Rahmen der Grunderkrankung bzw. einer zu forcierten diuretischen Therapie der Ödeme sind aber klinisch ernst zu nehmen. Die initiale Therapie sowie auch die Therapie im Verlauf richtet sich nach dem Schweregrad der Erkrankung.

## Diagnostik

Anamnestisch sollte auf Hinweise auf eine Systemerkrankung, Infektionen, Malignomen, Familienanamnese, z. B. Hinweis auf mögliche genetische Nierenerkrankung, sowie Medikamente geachtet werden.

Das Basis-Labor sollte neben den allgemein üblichen internistischen Serum-Parametern zusätzlich die Bestimmung der PLA2R-Ak, ANA, Komplement C3/C4 und bei Mikrohämaturie MPO-/PR3-Ak umfassen inkl. einer Hepatitis B und C Serologie. Bei negativen PLA2R-Ak sollten, wenn die Möglichkeit besteht, noch THSD7A-Ak bestimmt werden, aus dem Spontanharn ein Harnstix und Harnsediment sowie Protein/Kreatinin-Ratio. Initial empfehlen wir einmalig die Bestimmung eines 24-h-Sammelharns mit Kreatinin-Clearance und 24 h-Proteinurie um einen Ausgangswert zu haben und um abschätzen zu können, wie gut die Protein/Kreatinin-Ratio aus dem Spontanharn die tatsächliche 24 h-Proteinurie widerspiegelt.

Eine Tumorvorsorge ist altersentsprechend durchzuführen und fakultativ je nach Klinik, Familienanamnese und Risikoprofil entsprechend zu erweitern. Einen Abdomen-Ultraschall sowie ein Lungenröntgen (oder Thorax-CT) empfehlen wir bei jedem Patienten. Viele Zentren führen auch eine Bestimmung des Eisenstoffwechsels durch und empfehlen eine Teilnahme am nationalen Tumor-Screening-Programm für Mamma- und Colon-Karzinom, ein PSA-Test wird für alle Männer älter als 50 Jahre ebenso durchgeführt.

Der Großteil der MN kann mit dem Nachweis von PLA2R-Ak bewiesen werden (in ca. 70 % der Fälle bei primärer MN positiv) [3]. Für die Routinediagnostik zur Detektion von PLA2R-Ak sind derzeit zwei Assays von Euroimmun erhältlich, nämlich ein indirekter Immunofluoreszenz-Test (IIFT) sowie ein ELISA. Der IIFT hat eine gering höhere Sensitivität als der ELISA, bezogen auf den IIFT hat der ELISA eine Sensitivität von 96 %, die Spezifität ist bei beiden Tests praktisch gleich [4, 5].

Hinsichtlich Affektion der Sensitivität und Spezifität der Assays sowie mögliche Diskrepanzen zum immunhistochemischen Nachweis von PLA2R-Antigen in den Immunkomplexen in der Nierenbiopsie, ist vor allem der Zeitpunkt der PLA2R-Ak Bestimmung im Serum und Nierenbiopsie bezogen auf den Krankheitsverlauf sehr wichtig. Zum einem können anfangs die PLA2R-Ak auf Grund ihrer hohen Affinität zum PLA2R am Podozyten schnell und vollständig gebunden werden und somit unter der Nachweisgrenze im Serum liegen, sodass erst nach vollständiger Sättigung des PLA2R-Aks in der Niere PLA2R-Ak im Serum nachgewiesen werden können („*Kidney as a sink*“-Hypothese). Zum anderen können nach Sistieren der immunologischen Aktivität, bei Spontan-Remission oder nach Immunsuppression, Serum-PLA2R-Ak verschwunden sein, aber noch in der Nierenbiopsie nachweisbar sein, da dort der Abbauprozess der in Immunkomplexen gebundenen PLA2R-Ak deutlich länger dauert (siehe Abb. 5).

**Figure 2:**
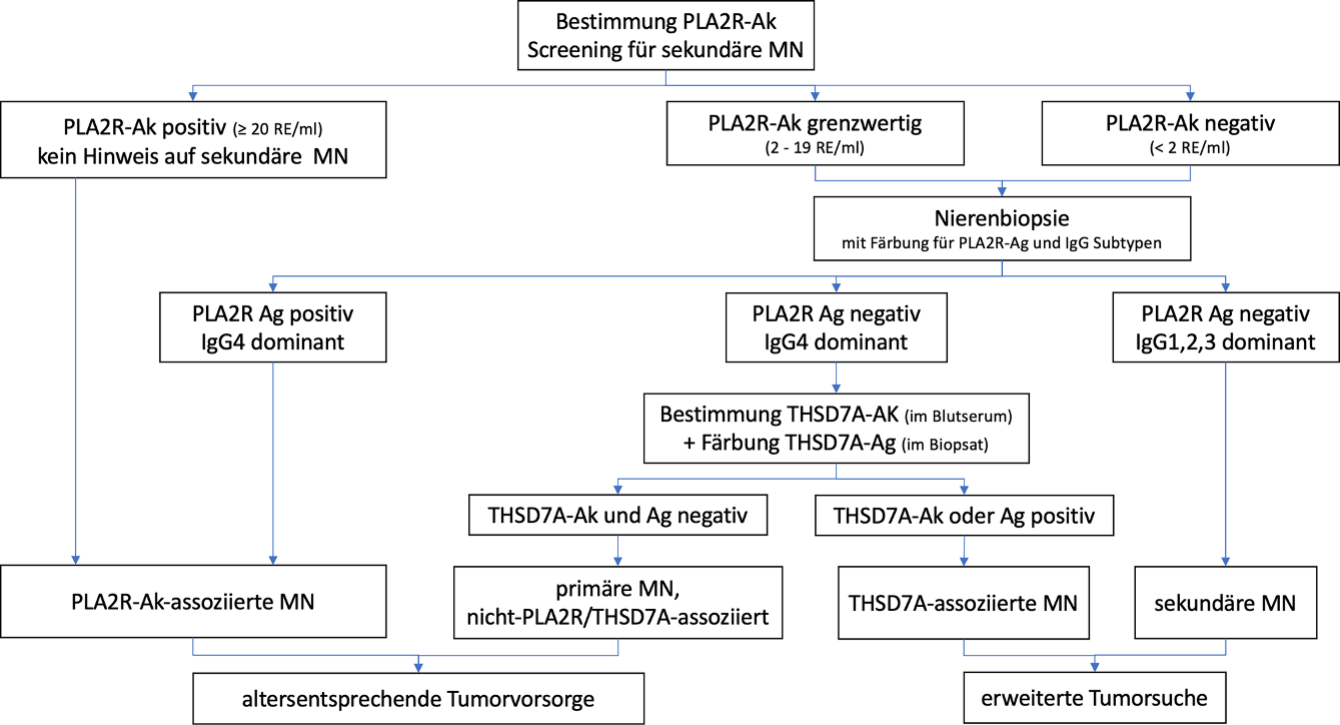

Auf eine diagnostische Nierenbiopsie kann verzichtet werden, wenn klare Positivität von PLA2R-Ak (> 20 RE/ml im ELISA von Euroimmun oder positiver IIFT) besteht, die Nierenfunktion weitgehend erhalten ist (eGFR > 60 ml/min/1,73 m^2^) und andere Ursachen für das klinische Bild ausgeschlossen sind (Abb. 2; [6]). Bei eingeschränkter Nierenfunktion sind einerseits auch die sehr seltene MN mit Halbmondbildung sowie andere Pathologien (bei entsprechender Komorbidität z. B. diabetische Nierenerkrankung) zu erwägen und deshalb eine Nierenbiopsie anzustreben.

Ideal wäre zur Diagnosesicherung eine Kombination von IIFT und ELISA (insbesondere bei ELISA Werten für PLA2R-Ak zwischen 2 und 20 RE/ml), jedoch benutzen nahezu alle Zentren in Österreich den ELISA Test, da ein IIFT nur semiquantitativ und untersucherabhängig ist. Bei einem PLA2R-Ak-Titer unterhalb von 20 RE/ml und fehlender Möglichkeit eines PLA2R-Ak IIFT ist eine Nierenbiopsie sinnvoll (PLA2R-Ag Nachweis in Immunkomplexen in der Nierenbiopsie?). Dennoch ist bei Negativität von PLA2R-Ak im Serum aber Ag-Positivität in der Nierenbiopsie eine weitere serielle Testung der PLA2R-Ak sinnvoll, da wie oben besprochen die Autoantikörper von Podozyten komplett abgesättigt werden können und ggf. erst im Verlauf auch im Serum nachweisbar werden [7].

Eine Positivität für PLA2R-Ak schließt andere Erkrankungen wie z. B. Neoplasien, Sarkoidose, Hepatitis B, SLE, etc. als mögliche Ursache für eine MN nicht gänzlich aus. Die in der Literatur beschriebenen Fälle lassen aber eher auf eine zufällige Koinzidenz einer primären PLA2R-Ak-assoziierten MN mit einer weiteren Erkrankung schließen, die Evidenz ist derzeit aber noch nicht ausreichend überzeugend, weshalb diese Möglichkeiten in der Diagnosestellung mitberücksichtigt werden sollten.

Insgesamt stellen PLA2R-Ak exzellente hochspezifische Biomarker dar, die sowohl die Diagnose in der Regel ohne Nierenbiospie sichern als auch die erfolgreiche Therapie inklusive Rezidiv im Krankheitsverlauf individualisiert ermöglichen [1].

Von Euroimmun ist auch ein IIFT für den Nachweis von THSD7A-Ak erhältlich, dieser hat aber in den meisten Zentren in Österreich in der Routinediagnostik noch keinen Einzug gefunden. Für die „neuen“ Antigene/Antikörper (siehe Abb. 1) sind noch keine kommerziellen Assays verfügbar, werden aber vermutlich in Zukunft ähnliche Bedeutung wie der PLA2R-Ak in Rahmen von Diagnostik, Klassifikation und Therapie der MN bekommen, insbesondere sind auch die Assoziationen dieser Ag/Ak mit anderen immunologischen Erkrankungen oder Malignomen zu beachten.

**Figure 3:**
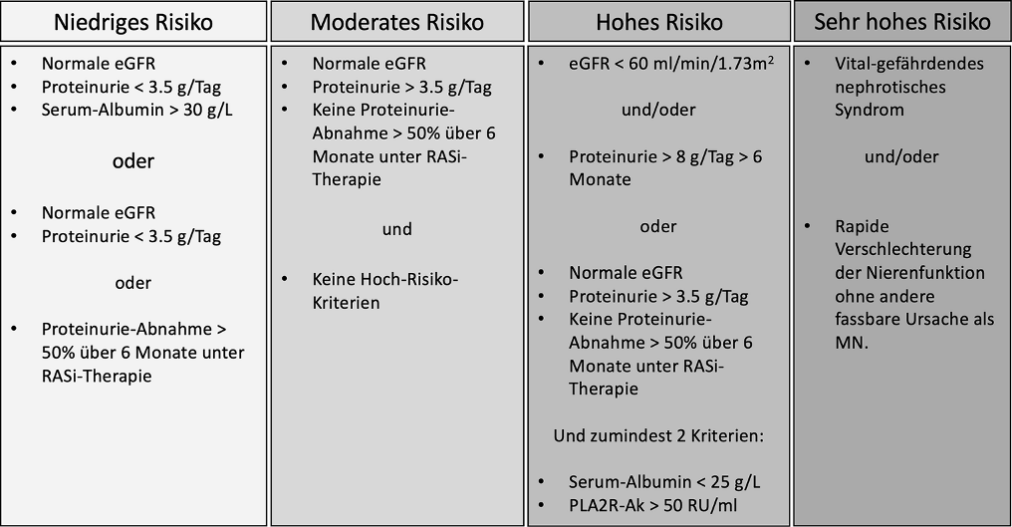

Entsprechend den rezenten KDIGO Guidelines kann u. a. mit Erhalt der PLA2R-Ak-Titer eine weitere Risikostratifizierung nach MN Diagnose erstellt werden (siehe Abb. 3; [8]): es muss jedoch betont werden, dass bislang keine prospektive Evaluation dieser Kategorisierung im Sinne eines prädiktiven Scores durchgeführt worden ist. Zudem weicht unser empfohlenes therapeutisches Vorgehen von dieser Klassifizierung ab (s. unten).

## Therapie

Initial steht die nicht-immunsuppressive Therapie der Proteinurie und/oder des nephrotischen Syndroms im Vordergrund. Neben einer RAS-Blockade ist v. a. eine Therapie mit einem SGLT-2-Inhibitor anzustreben. Bei Vorliegen einer Hyperlipidämie sollten Statine eingesetzt werden, bzw. alternativ PCSK9-Hemmer bei Statin-Unverträglichkeit oder unzureichender therapeutischer Wirksamkeit. Darüber hinaus sind Diuretika bei Ödemen im Rahmen des nephrotischen Syndroms meist erforderlich. Auf Grund des erhöhten Thromboembolie-Risikos kann insbesondere bei ausgeprägter Hypalbuminämie eine prophylaktische Antikoagulation nach individuellem Risiko-Nutzen-Verhältnis erwogen werden. Hier ist die Dosierung entsprechend einer therapeutischen Antikoagulation bei Thromboembolie vorzuziehen (Verweis: Separater Beitrag „Allgemeine Empfehlungen für die Behandlung glomerulärer Erkrankungen“).

Eine immunsuppressive Therapie der MN ist grundsätzlich indiziert, wenn ein nephrotisches Syndrom vorliegt und es 6–9 Monate nach Auftreten der ersten klinischen Symptome und/oder Labor-Befunde nicht zu einer Spontanremission gekommen ist. Bei sehr hohen Antikörper-Titern und bestehendem nephrotischen Syndrom kann nach eigenem Ermessen auch sofort eine immunsuppressive Therapie begonnen werden.

Wir empfehlen einen sofortigen Start einer Immunsuppression bei raschem Nierenfunktionsverlust auf Basis der MN, oder auch wenn ernsthafte Komplikationen durch das nephrotische Syndrom auftreten, sowie auch bei sehr hohem Leidensdruck.

**Figure 4:**
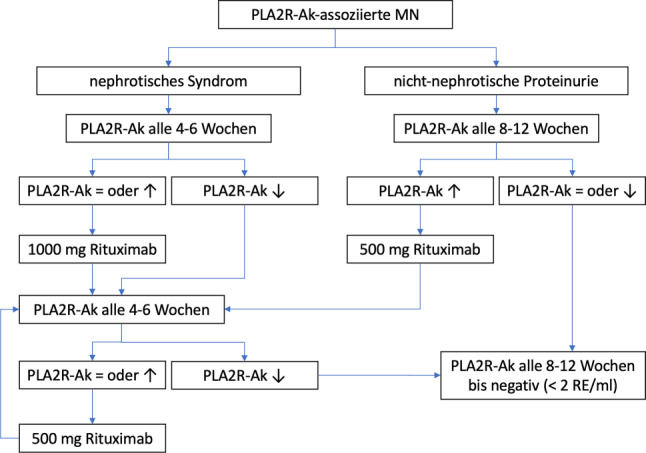

Bei PLA2R-Ak-assoziierter MN empfehlen wir ein Vorgehen je nach Verlauf des PLA2R-Ak-Titers (siehe Abb. 4). Nachdem diese Empfehlung jedoch nicht strikt evidenz-basiert ist und von den KDIGO-Empfehlungen abweicht, sollte unbedingt eine ausführliche Information und eine genaue Nutzen-Risikoabwägung im Sinne eines „*informed decision making*“ mit dem Patienten erfolgen [7].

Eine Immunsuppression ist nicht indiziert bei Vorliegen einer Kontraindikation wie z. B. einem floriden Infekt, bei geringer bzw. nicht-nephrotischer Proteinurie, bei PLA2R-Ak-negativer primärer MN, sowie nicht bei einer hochgradigen chronischen Nierenschädigung mit einer persistierenden eGFR < 30 ml/min/1,73 m^2^, einem Hinweis auf signifikante Nierenparenchym-Schädigung in der Bildgebung und/oder in Histologie hochgradige interstitielle Fibrose.

Als immunsuppressive Substanzen werden Rituximab als Monotherapie sowie Cyclophosphamid und Kalzineurin-Inhibitoren, meist in Kombination mit unterschiedlichen Dosen von Kortikosteroiden eingesetzt. Eine Monotherapie mit Kortikosteroiden ist bei der MN wirkungslos.

In rezenten prospektiven Studien zur immunsuppressiven Therapie (u. a. GEMRITUX, MENTOR, STARMEN, RI-CYCLO), betrug sechs Monate nach Therapiebeginn die Rate des Ansprechens ca. 70–90 %, jedoch sind die Studien aufgrund variabler Einschlusskriterien, Therapieintensität sowie kurzer Beobachtungsdauer kaum miteinander vergleichbar und auf Grund der inhomogenen bzw. problematischen Studiendesigns nicht geeignet eine Evidenz betreffend einer Über- oder Unterlegenheit von Rituximab oder Cyclophosphamid zu liefern [9–11]. Cyclophosphamid und insbesondere Rituximab führen zu einer Unterdrückung der B‑Zellen und konsekutiv auch zu einer verminderten Bildung der pathogenetischen Auto-AK, wohingegen Kalzineurin-Inhibitoren vermutlich vor allem durch Beeinflussung des Podozyten-Zytoskeletts zur Proteinurie-Reduktion führen (deshalb nach Absetzen regelhaft rasche Rezidive).

Wenn eine Immunsuppression indiziert ist, empfehlen wir primär eine Monotherapie mit Rituximab, da Rituximab das günstigste Wirkungs‑/Nebenwirkungs-Verhältnis aufweist und zudem selektiver die Autoantikörperproduktion verhindert [12, 13].

Es gibt verschiedene Dosierungsschemen von Rituximab, eine gute Evidenz für ein bestimmtes Schema gibt es jedoch nicht. Aus allen Studien ist zumindest ableitbar, dass die erste Rituximab-Dosis nicht zu niedrig gewählt werden sollte, insbesondere bei großer Proteinurie, da Rituximab hier vermehrt renal verloren geht (und deshalb oftmals auch eine weitere Rituximab-Gabe erforderlich zu sein scheint). Wir schlagen deshalb bei nephrotischem Syndrom initial die Gabe von 1000 mg Rituximab vor, bei nicht-nephrotischer Proteinurie kann mit 500 bis 1000 mg Rituximab begonnen werden. Die weitere Dosierung von Rituximab richtet sich dann bei der PLA2R-Ak-assoziierten MN nach der PLA2R-Ak-Dynamik mit dem Ziel, diese vollständig zu depletieren (d. h. < 2 RE/ml im Euroimmun ELISA, siehe Abb. 4; [1, 9]).

Bei PLA2R-Ak-negativer MN fehlt leider ein guter Serumparameter, um die Effektivität der Immunsuppression zu monitieren. Wir schlagen deshalb vor, bei PLA2R-Ak-negativer MN innerhalb der ersten zwei Wochen nach der initialen Verabreichung von 1000 mg Rituximab B‑Zellen mit Durchflusszytometrie/Leukozytentypisierung die CD19 positiven Zellen quantitativ im Blut zu bestimmen, um über die B‑Zell-Depletion einen indirekten Hinweis zur Effektivität der Therapie zu bekommen. Bei einem Messwert ≥ 5/mm^3^ empfehlen wir eine weitere Rituximab-Gabe mit 500 mg; dieses Vorgehen kann wiederholt werden bis zu einer maximalen Dosis von 2000 mg Rituximab innerhalb von 4 Wochen. Je nach klinischem Verlauf der Proteinurie können nach jeweils 6 Monaten nochmals 500–1000 mg Rituximab verabreicht werden. Auch hier können ggf. die B‑Zellen einen Hinweis auf eine suffiziente Dosierung geben, aber B‑Zellen korrelieren generell *per se* nicht gut mit dem Krankheitsverlauf und ihre Bestimmung dient daher nur als Hilfsbefund zur suffizienten Rituximab-Dosierung zu werten.

Die Indikation für eine Therapie mit Cyclophosphamid sehen wir nur in Einzelfällen, z. B. Patientenwunsch, Unverträglichkeit von oder Nebenwirkungen durch Rituximab (z. B. ausgeprägte Hypogammaglobulinämie), oder wenn zunächst eine Impfung abgewartet werden sollte, da nach Rituximab unter B‑Zell Depletion mit einerm schlechten/fehlenden Impfansprechen gerechnet werden muss. Dies ist insbesondere relevant bei z. B. COVID 19. Cyclophosphamid stellt auch eine Alternative bei nicht-Ansprechen auf Rituximab dar, wobei da genau zu evaluieren wäre, ob tatsächlich noch eine immunologische Aktivität vorliegt oder eine persistierende Proteinurie auf einen irreversiblen/chronischen glomerulären Schaden zurückzuführen ist, oder, ob Rituximab unterdosiert war. Sollte Cyclophosphamid zum Einsatz kommen, so besteht die Möglichkeit der Verabreichung des a) modifizierten Ponticelli-Regimes (2,5 mg/kg/KG/Tag zyklisch) oder b) des niederländischen Protokolls mit Cyclophosphamid p.o. mit 1,5–2 mg/kg/KG/Tag für 12 Monate gemeinsam mit Steroid-Puls-Therapie i.v. alle ein, drei und fünf Monate sowie c) Cyclophosphamid-Therapie 15 mg/kg/KG i.v. alle drei Wochen für insgesamt 6 Monate ebenso mit Steroid-Puls-Therapie.

Sollte eine Unverträglichkeit von Rituximab bestehen oder es zu einem tatsächlichen Therapieversagen von Rituximab kommen, wäre alternativ auch eine Therapie mit einem neueren CD20-Antikörper zu erwägen, z. B. Obinutuzumab, bisher sind aber nur Fallberichte in der Therapie der MN publiziert, eine Phase 3 Studie ist aktuell laufend [11].

Die Indikation für eine Therapie mit einem Kalzineurin-Inhibitor sehen wir im Gegensatz zur KDIGO Guideline (wobei die KDIGO Empfehlung auf Grund der vorhandenen Evidenz schwer nachvollziehbar ist) als noch limitierter als für Cyclophosphamid. Der Einsatz von Kalzineurin-Inhibitoren kann in speziellen Situationen Sinn machen, wie z. B. Proteinurie-Reduktion bis zum Start einer Anti-B-Zell-Therapie, der generelle Wunsch einer raschen Proteinurie-Reduktion bei gleichzeitigem Start mit Rituximab (Hybrid-Therapie) bei schwerem nephrotischen Syndrom, wenn Kontraindikationen sowohl gegen eine Therapie mit Rituximab als auch eine Therapie mit Cyclophosphamid bestehen bzw. diese vom Patienten abgelehnt werden. Zwei prinzipielle Gründe limitieren jedoch generell den Einsatz von Kalzineurin-Hemmern bei MN: 1) die bekannte Nephrotoxizität im chronischen Verlauf und 2) die konsistent hohe Rezidivrate nach Absetzen der Therapie. Als Kalzineurin-Inhibitoren können sowohl Cyclosporin A als auch Tacrolimus verwendet werden, mit Zielspiegeln von 80–175 ng/ml bzw. 5–8 ng/ml.

**Figure 5:**
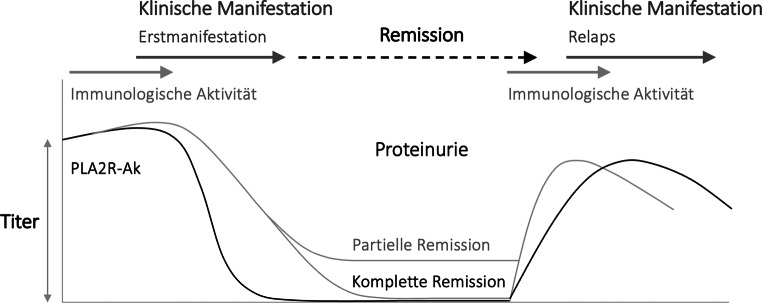

Bei primärer MN ist grundsätzlich bei jeder immunsuppressiven Therapie der zeitlich versetzte Verlauf der immunologischen und klinischen Aktivität zu beachten (Abb. 5), da nach immunologischer Remission oft noch viele Monate bis Jahre vergehen können, ehe die abgelagerten Immunkomplexe abgebaut und die betroffenen Glomeruli wieder komplett rekonstituiert sind.

Nach erfolgter Therapie kann zwischen einer partiellen und einer kompletten klinischen Remission (> 50 % Reduktion der Ausgangsalbuminurie und > 90 % Reduktion der Albuminurie oder < 0,5 g Albuminurie/Tag) unterschieden werden.

Prinzipiell ist das therapeutische Ziel ein komplettes immunologisches Ansprechen, welches bei PLA2R-Ak-assoziierter MN vorliegt, wenn die PLA2R-Ak nicht mehr nachweisbar sind (d. h. < 2 RE/ml im ELISA von Euroimmun). Selbst bei niedrigen PLA2R-Ak Titern ist von einer kontinuierlichen Ablagerung in den Podozyten und damit Krankheitsaktivität (mit persistierender Proteinurie) auszugehen.

Die Entdeckung der PLA2R-Ak hat das Vorgehen bei MN revolutioniert, da diese bei Positivität diagnostisch sichernd sind und auch das weitere therapeutische Vorgehen bestimmen, sodass eine hochindividuelle Therapie möglich ist. Zum einen weist ein im Verlauf fallender PLA2R-Ak Titer auf eine Spontanremission hin, zum anderen kann eine immunsuppressive Therapie nach PLA2R-Ak Verlauf adaptiert werden. Dies ist insofern von großer Wichtigkeit, da die immunologische Aktivität (PLA2R-Ak Bildung) und die klinische Manifestation (Proteinurie) zeitverzögert sind (Abb. 5). Des weiteren kann es im Krankheitsverlauf zu irreversiblen glomerulären Strukturalterationen gekommen sein, welche zu einer persistierenden Proteinurie bei fehlender immunologischer Aktivität führen.

In allen Fällen einer immunologischen Remission gilt, dass der Patient nicht mehr von einer Immunsuppression profitiert, so dass diese beendet bzw. gar nicht erst begonnen werden sollte. Bei der PLA2R-Ak-negativen primären MN besteht auf Grund des fehlenden Nachweises vom PLA2R-Ak oder anderer Autoantikörper leider nicht die Möglichkeit, die immunologische Aktivität mit einem Serummarker zu beurteilen, sodass nur über den klinischen Verlauf Rückschlüsse auf die Krankheitsaktivität möglich sind. Im Einzelfall sollte bei Unklarheiten, z. B. bei lange persistierender signifikanter Proteinurie, zur DD immunologische Aktivität vs. irreversible glomeruläre/tubuläre Strukturalteration, eine Nierenbiopsie im Verlauf erwogen werden.

## Follow-Up

Die Nachsorge der Patienten entspricht dem üblichen Vorgehen bei Patienten mit vergleichbarer Proteinurie bzw. Nierenfunktionseinschränkung.

Bei PLA2R-Ak-assoziierter MN empfehlen wir zusätzlich die Bestimmung der PLA2R-Ak alle 4–12 Wochen (siehe Abb. 4) bis diese sicher negativ sind (d. h. < 2 RE/ml), danach alle drei Monate für zumindest die nächsten 2–3 Jahre. Anschließend kann einmal pro Jahr eine Ak-Bestimmung im Rahmen einer Kontrolle an z. B. einem nephrologischen Zentrum erfolgen.

Bei PLA2R-Ak-negativer MN sollten PLA2R-Ak, wenn keine immunsuppressive Therapie erfolgt, noch einmal nach drei Monaten bestimmt werden.

Bei jedem Relaps einer MN sollten PLA2R-Ak bestimmt werden.

Bei Immunsuppression, insbesondere Rituximab-Gabe, sollten die Immunglobuline alle sechs Monate kontrolliert werden, bis diese wieder im Normalbereich sind. Bei Hepatitis-B-Anamnese empfehlen wir zusätzlich Kontrollen der „Leberwerte“ (GPT, GGT, AP, Bilirubin) sowie eine HBV-PCR alle drei Monate bis ein Jahr nach Beendigung der Immunsuppression, da eine HBV-Reaktivierung unter immunsuppressiver Therapie möglich ist. Auch im Follow-Up ist eine altersentsprechende Tumorvorsorge zu beachten.
